# Long-term effectiveness of cognitive behavioral therapy (CBT) for children and adolescents in routine care

**DOI:** 10.1186/s40359-026-04667-3

**Published:** 2026-05-06

**Authors:** Matthis Michael Hüwelmeier, Lena Staniczek, Silvia Schneider, Xiao Chi Zhang, André Wannemüller, Gerrit Hirschfeld, Karen Krause, Sören Friedrich, Ruth von Brachel

**Affiliations:** 1 Mental Health Research and Treatment Center (FBZ), Ruhr University Bochum, Bochumer Fenster Massenbergstraße 9-13, Bochum, 44787 Germany; 2 German Center for Mental Health (DZPG), partner site Bochum-Marburg, Bochum, Germany; 3 Faculty of Business and Health, University of Applied Sciences Bielefeld, Bielefeld, Germany; 4 Department of Psychology, University of Freiburg, Freiburg, Germany

**Keywords:** CBT, Children, Adolescents, Routine care, Long-term effectiveness, Cross-diagnostic

## Abstract

**Background:**

Cognitive behavioral therapy (CBT) is one of the most widely established treatments for mental disorders in children and adolescents and is empirically supported across a wide range of disorders, including evidence from routine care. However, evidence on long-term maintenance of effects in routine outpatient care is still limited, particularly across diagnostic groups. This study examines the long-term, cross-diagnostic effectiveness of CBT in children and adolescents treated under routine outpatient care conditions.

**Methods:**

Analyses are based on pre-existing routine outcome monitoring data from 1225 patients (mean age = 14.00 years, SD = 3.24) receiving CBT, collected between 2017 and 2025. Symptoms were assessed using the parent- and patient-reported Strengths and Difficulties Questionnaire (SDQ) at pre-treatment, post-treatment and at 6-, 12- and 24-month follow-ups. Additional self-rated treatment-success ratings were collected at follow-up (covering expectations fulfilled, perceived helpfulness, problem recurrence and perceived change). Effectiveness was described using group means as well as clinical significance. Pre- to post- and pre- to follow-up changes were analysed using paired Wilcoxon signed-rank tests. Associations between follow-up SDQ scores and self-rated treatment success were examined using partial Spearman correlations controlling for baseline SDQ.

**Results:**

Across both parent- and patient-reported SDQ assessments, total difficulties and problem subscales showed significant improvements from pre- to post-treatment and from pre-treatment to all follow-up time points. Effect sizes were consistently moderate-to-large *(*r_rb = 0.59–0.77), with sustained effects up to 24 months. Higher follow-up SDQ difficulties were associated with lower self-rated treatment success across multiple follow-up ratings, with strongest associations observed for problem recurrence and change compared to before treatment. Sensitivity analyses restricted to participants in the clinical range at baseline (SDQ ≥ 17) yielded consistent results.

**Conclusions:**

Routine outpatient CBT for children and adolescents was associated with meaningful symptom improvements that persisted up to two years after treatment. Clinically significant change analyses indicated that while many patients showed reliable improvement, a substantial proportion remained classified as unchanged according to conservative criteria. Subjective follow-up ratings were consistent with standardized symptom outcomes, supporting the perceived durability of treatment success under routine care conditions in a diagnostically heterogeneous sample.

**Trial registration:**

Not applicable. This study is an observational analysis of routinely collected data. The analysis plan was preregistered at PsychArchives (10.23668/psycharchives.21444).

**Supplementary Information:**

The online version contains supplementary material available at 10.1186/s40359-026-04667-3.

## Background

Cognitive behavioral therapy (CBT) has established itself as a successful psychotherapy method for a wide range of disorders in children and adolescents; its efficacy has been demonstrated in various reviews and meta-analyses [1–3]. According to national health insurance data from Germany, 1.4% of all children and adolescents with statutory health insurance in Germany received regular psychotherapy in 2019, with 56.7% of these receiving CBT [4].

Across existing studies, findings on CBT outcomes in children and adolescents differ substantially depending on several key design characteristics, including whether studies were conducted under efficacy or effectiveness conditions, whether they focused on single diagnostic groups or heterogeneous samples, which informants were used to assess outcomes (parent- vs. patient-reported measures) and whether outcomes were examined over short-term or longer-term follow-up periods. With regard to study design, while most efficacy studies used strictly guided manuals and highly selected patients, an increasing body of evidence suggests that CBT is also effective when delivered in routine care settings across more diverse patient samples. Systematic reviews and meta-analyses indicate that CBT under routine care conditions is associated with symptom improvements in children and adolescents. In a comprehensive meta-analysis, Wergeland, Riise and Öst (2021) summarized 58 routine care studies including 4618 young patients with anxiety, depression, obsessive-compulsive disorder or post-traumatic stress disorder and reported large pre- to post-treatment effects as well as maintained improvements at follow-up. Follow-up periods varied substantially across studies, ranging from short-term assessments to several years after treatment, with most studies focusing on follow-ups within the first year [5]. Similarly, a recent review of randomized controlled trials conducted in routine settings found small but significant effects of psychotherapy on anxiety and depressive symptoms compared to non-active controls in children and adolescents [6].

Despite this growing evidence base, follow-up assessments in routine care studies are often limited to relatively short periods and long-term maintenance of treatment effects remains insufficiently investigated. Long-term follow-up data in naturalistic outpatient settings in children and adolescents have been reported only in a small number of studies and are largely restricted to specific diagnostic groups. For instance, long-term maintenance of treatment effects has been demonstrated in a single study with an average follow-up of 5.3 years, although this study focused exclusively on anxiety and depressive disorders [7]. Additional evidence for sustained effects comes from a naturalistic outpatient study of children and adolescents with anxiety disorders, in which remission rates and functional improvements remained stable more than four years after CBT delivered under routine conditions [8]. Comparable long-term findings have also been reported in adult samples treated in routine outpatient care across various disorders [9]. 

In the context of routine outpatient care in Germany, Walter et al. examined changes in emotional and behavioral problems among adolescents receiving CBT in a university-based outpatient clinic [10]. Significant and partly medium-to-large symptom reductions were observed, although a substantial proportion of patients remained within the clinical range at treatment end. Interpretation of these findings is limited by the absence of long-term follow-up assessments. A related study by the same research group reported similar pre- to post-treatment improvements in completer analyses, but likewise did not include follow-up data [11].

Taken together, existing research supports the effectiveness of CBT delivered under routine care conditions. However, evidence on the long-term maintenance of treatment effects remains limited, particularly in diagnostically heterogeneous samples and in studies that systematically integrate both patient- and parent-reported outcome measures.

Across available studies, follow-up assessments were mostly limited to 12 months or less, allowing only restricted conclusions about the longer-term maintenance of treatment gains. In addition, studies conducted under routine care conditions differ substantially with regard to patient characteristics, diagnostic focus and treatment delivery, which limits the comparability of findings across samples and disorders [5, 6].

Despite robust evidence for the efficacy of manualized CBT for specific disorders and the demonstrated effectiveness of CBT in routine care, particularly for depressive and anxiety symptoms, evidence for externalizing problem domains remains comparatively limited.

Against this background, cross-diagnostic investigations with long-term follow-up in naturalistic outpatient settings remain scarce. In particular, the sustainability of treatment effects beyond the end of therapy has received comparatively little attention. To our knowledge, no study has yet examined the long-term, cross-diagnostic effectiveness of CBT in routine outpatient care for children and adolescents. In the present study, the term cross-diagnostic refers to a diagnostically heterogeneous sample reflecting routine outpatient care, rather than analyses comparing specific diagnostic groups.

Beyond symptom change, studies indicate that improvements in standardized outcome measures are typically accompanied by patients’ and caregivers’ subjective perceptions of benefit and recovery. While standardized symptom measures capture changes in psychopathology, subjective evaluations reflect appraisals of treatment benefit and everyday functioning, addressing overlapping but distinct aspects of outcome [12, 13]. Examining the correspondence between standardized symptom outcomes and subjective evaluations in both patients’ and caregivers’ ratings therefore represents an important indicator of perceived treatment effectiveness in routine care.

### Objectives and hypotheses

At the Research and Treatment Centre for Mental Health in Bochum, Germany, children and adolescents are surveyed about their current state of health and the success of their therapy at 6, 12 and 24 months after completion of routine therapy (CBT), in addition to examinations at the pre- and post-treatment stages. Our aim is to quantitatively evaluate the cross-diagnostic effectiveness of CBT in routine care using these data. The effectiveness will be described both in terms of group-mean changes as well as clinical significance [14] in order to evaluate whether these changes are also meaningful from an individual participant’s point of view.

H1) CBT delivered in routine care is effective in a diagnostically heterogeneous sample.

H2) Therapeutic improvements persist 6, 12 and 24 months after treatment completion.

H3) Higher symptom severity at follow-up (SDQ) is associated with less favorable self-rated treatment success at follow-up.

## Methods

### Aim, design and setting

This was an observational study with repeated measures based on routine outcome monitoring data from both patients and caregivers (predominantly parents) at the Research and Treatment Centre for Mental Health in Bochum, Germany (2017–2025). Assessments were usually completed on computers on site after therapy sessions. Follow-up examinations were voluntary and conducted on site whenever feasible or remotely.

### Participants

Participants were patients receiving CBT carried out under supervision. Data collection was part of routine outcome monitoring, no additional recruitment or compensation took place. The sample comprised *N* = 1225 patients (mean age at baseline: M = 14.00 years, SD = 3.24, range 5.1–20.9). Sample size varies across analyses due to missing questionnaire data at specific assessment points. The maximum age at assessment reflects routine outpatient care practices in Germany, where child and adolescent psychotherapists are licensed to treat patients up to the age of 21 and psychotherapeutic treatment may be initiated or continued beyond age 18.

CBT was delivered under routine outpatient care conditions in a supervised university training centre. Treatment was based on core CBT principles (e.g., cognitive restructuring and behavioral techniques) and was individualized according to patients’ presenting problems, consistent with routine care practice. Due to the naturalistic design and the use of routinely collected data, detailed information on specific treatment components or the exact number of sessions was not systematically recorded. The mean time between pre- and post-treatment assessment was 12.33 months (SD = 7.40, range = 0.59–49.30 months). In the German routine outpatient care system, CBT typically involves approximately 12–60 sessions depending on severity and treatment phase, which provides contextual information regarding treatment intensity. Therapists were in their second or third year of a three-year postgraduate training program in CBT and received regular and intensive supervision (approximately every fourth session) by licensed CBT practitioners.

The sample was diagnostically heterogeneous, as typically observed in routine outpatient care. At pre-treatment, anxiety and stress-related disorders (including adjustment disorders) were the most frequently documented diagnostic category (53.5%), followed by behavioral and emotional disorders (33.4%) and mood disorders (26.9%). Developmental disorders, psychotic disorders, substance-related disorders and other mental disorders were less common. Because multiple diagnostic categories could be documented for a single patient, percentages do not sum to 100%. Overall, 25.0% of the patients had diagnoses spanning more than one ICD-10 diagnostic category.

Diagnoses were assigned by clinicians using ICD-10 criteria as part of routine care. At the centre, clinicians are routinely trained in the use of structured diagnostic interviews (e.g., Kinder-DIPS [15]) and standard operating procedures include conducting diagnostic interviews with the child and at least one caregiver. However, the specific use of structured interviews was not systematically documented within the routine outcome monitoring dataset.

Information on pharmacological treatment (e.g., medication for ADHD, anxiety or depression) was not systematically recorded within the routine outcome monitoring dataset and was therefore not available for analysis.

### Inclusion/exclusion (post-data collection)

Participants were excluded if no valid SDQ data were available at pre-treatment or post-treatment or if they had participated in other intervention studies conducted at the same centre that involved additional study-specific procedures and that are published separately (e.g., “Kids Beating Anxiety (KibA)” [8]). The present analyses were based on routinely collected outcome monitoring data. Data collection was conducted within the context of the KODAP research network, a collaborative initiative of university outpatient psychotherapy clinics in Germany aimed at improving routine outcome monitoring and practice-based research [16, 17].

### Primary outcome

Symptoms were assessed using the Strengths and Difficulties Questionnaire (SDQ), a widely used screening instrument with well-established psychometric properties in child and adolescent samples. The SDQ has been applied in both clinical and routine care settings across a broad range of diagnostic groups and has demonstrated adequate psychometric properties in heterogeneous child and adolescent samples. Analyses focused on the total difficulties score and five subscales (emotional symptoms, conduct problems, hyperactivity/inattention, peer problems and prosocial behavior) [18]. Parent- and patient-reported versions were analysed separately. The SDQ total difficulties score was defined as the primary outcome, while subscale scores were treated as secondary outcomes. In the present sample, internal consistency of the SDQ Total Difficulties score at pre-treatment was good in both patient (Cronbach’s α = 0.77) and parent reports (α = 0.79).

### Follow-up ratings

At follow-up, additional self-rated treatment-success ratings were collected using ordinal items slightly modified from Michalak et al. [12]. These included ratings of expectations fulfilled (0–5; FU6 only), perceived helpfulness (0–5; FU6 only), recurrence of initial problems (0–5), perceived change compared to before treatment (0–6) and perceived change compared to the end of treatment (0–6). For items assessing recurrence of initial problems and perceived change, higher scores indicate less favourable outcomes. Items assessing expectations fulfilled and perceived helpfulness were reverse-coded so that higher scores likewise indicated less favourable outcomes, ensuring a consistent direction across all items. Although follow-up ratings were measured on ordinal scales, descriptive statistics are reported as means and standard deviations for ease of interpretation; all inferential analyses appropriately relied on rank-based correlations and ordinal regression models.

### Other variables

Information on diagnosis, gender and age was extracted as secondary variables.

### Data handling, preprocessing, missing data

SDQ scoring followed standardized procedures. Data were screened for plausibility and implausible values were set to missing. All analyses were conducted using available cases at each assessment point. Given the non-random and structurally decreasing nature of missingness in routine follow-up assessments, no imputation was applied. Sample sizes varied across analyses due to assessment-specific missing data. Pre-post analyses included all participants with valid data at both time points, whereas follow-up analyses were conducted separately for each follow-up assessment using available cases.

Although the preregistered analysis plan specified data collection from 2019 onwards, eligible routine outcome monitoring data from 2017 to 2018 were additionally included because they were collected using identical assessment procedures, instruments and data management systems and were available at the time of analysis. This extension does not affect the preregistered hypotheses, analytic strategy or inference criteria.

### Statistical analyses

Pre- to post-treatment and pre-treatment to follow-up changes were analysed using paired Wilcoxon signed-rank tests (two-sided, α = 0.05). Clinically significant change was additionally classified using established reliable and clinically significant change criteria applied to SDQ total difficulties scores. Effect sizes were quantified using rank-biserial correlations derived from paired Wilcoxon signed-rank tests, reflecting the magnitude of within-subject change without the use of a binary grouping variable. Associations between follow-up SDQ scores and ordinal treatment-success ratings were examined using partial Spearman correlations controlling for baseline SDQ scores. Sensitivity analyses included Kendall’s tau-b correlations and ordinal regression analyses (cumulative link models) predicting follow-up ratings from SDQ change scores while controlling for baseline SDQ. For descriptive interpretation, rank-biserial correlations of approximately 0.10, 0.30 and 0.50 were considered small, medium and large effects. Exploratory analyses examined whether pre-post changes in SDQ total difficulties differed between ICD-10 diagnostic categories using Kruskal-Wallis tests. Additionally, sensitivity analyses were conducted restricting the sample to participants with SDQ total difficulties scores in the clinical range at baseline (≥ 17). All analyses were conducted in R. Clinically significant change was evaluated using the clinicalsignificance package [19]. Reliable change was calculated following the approach by Jacobson and Truax (1991). Reliability estimates were based on internal consistency (Cronbach’s α) of the SDQ total difficulties score at baseline (α = 0.77 for patient report and α = 0.79 for parent report). Classification into outcome categories followed combined criteria, including both reliable change and movement relative to the SDQ clinical cutoff. Effect sizes and confidence intervals were computed using effectsize [20] and ordinal regression models were estimated using the ordinal package [21]. Data handling and visualization were performed using standard R packages (e.g., tidyverse, ggplot2). Clinically significant improvement was defined as the combination of reliable change and movement from the clinical to the non-clinical range based on the SDQ cutoff.

## Results

### Data completeness

**Fig. 1:**
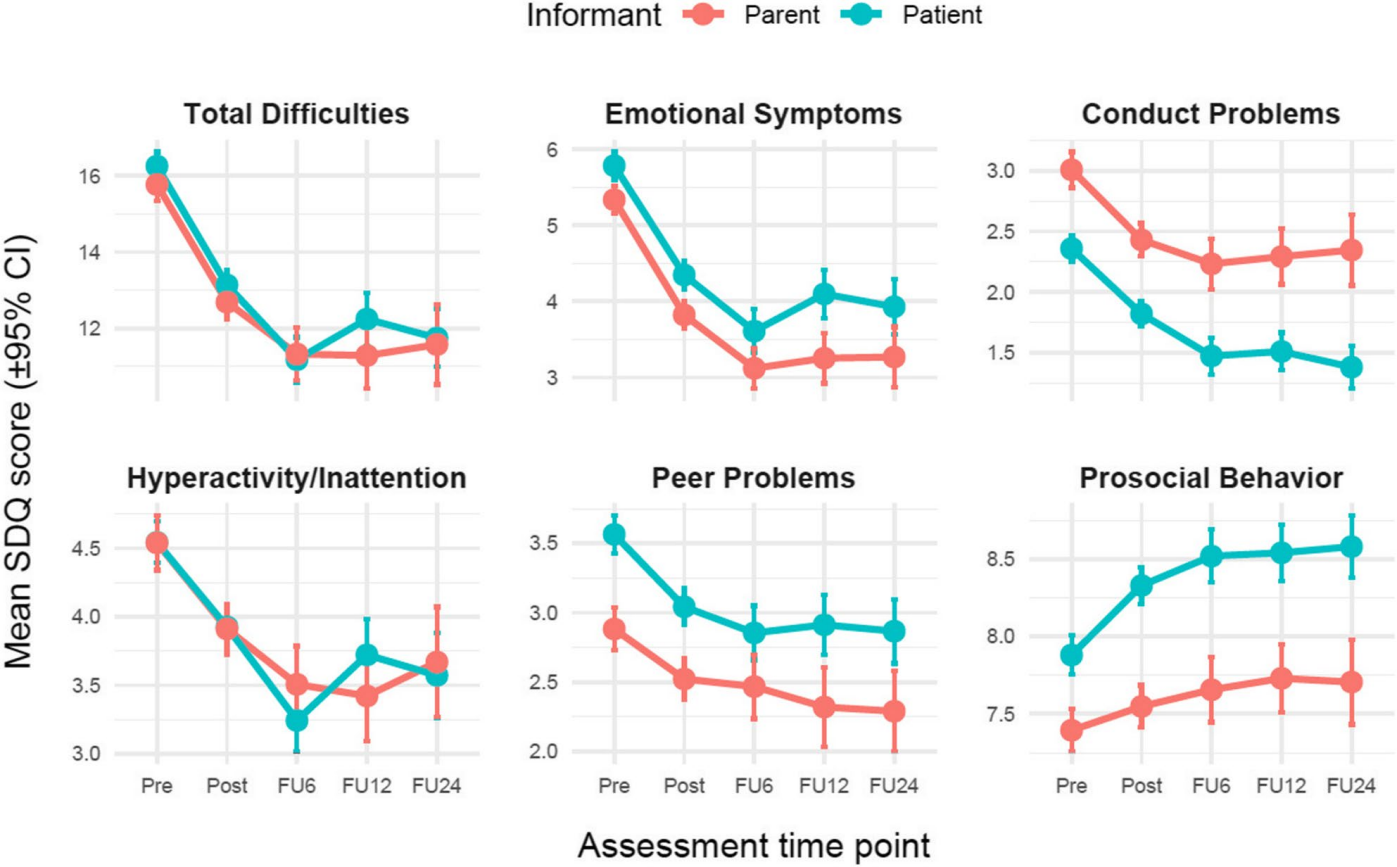
Mean SDQ scores by assessment time point and informant. Note. Descriptive means are shown separately for parent- and patient-reported outcomes at pre-treatment, post-treatment and 6-, 12-, and 24-month follow-ups. Values are based on analysis-specific subsamples available at each assessment point; sample composition therefore differs across time points due to selective follow-up participation. Higher scores on problem scales indicate greater difficulties, whereas higher scores on the prosocial scale indicate more prosocial behavior

Baseline SDQ data were available for all patients and for the majority of caregivers, resulting in slightly different baseline sample sizes across informants. As is typical for routine outcome monitoring, participation declined at follow-up assessments. The reduction in sample size was most striking at the 24-month follow-up. Dropout across follow-up assessments was substantial and is typical for routine outcome monitoring studies, particularly at longer follow-up intervals. All analyses were conducted using available cases at each assessment point. Sample sizes for patient and parent reports at each time point are shown in Table 1. Figure 1 illustrates the descriptive trajectories of parent- and patient-reported SDQ scores across assessment time points.

**Table 1:** Data completeness across assessment points

Informant	Pre	Post	FU6	FU12	FU24
Parent	1169	761	334	266	190
Patient	1225	881	363	319	254

Sample sizes for patient and parent reports at each time point. FU = follow-up (6, 12, 24)

### Pre- to post-treatment changes (H1)

Both patient- and parent-reported SDQ scores showed significant changes from pre- to post-treatment. For both informants, the total difficulties score decreased significantly, as did all problem-related subscales. Analysis-based descriptive statistics for SDQ scores are provided in Supplementary Table S1. Effect sizes were predominantly moderate to large for total difficulties and emotional symptoms and small to moderate for conduct problems, hyperactivity/inattention and peer problems (see Table 2). In patient reports, the largest reductions were observed for total difficulties and emotional symptoms. Conduct problems, hyperactivity/inattention and peer problems decreased significantly, prosocial behavior increased significantly over the course of treatment. Parent reports showed a similar pattern. Total difficulties and emotional symptoms decreased clearly from pre- to post-treatment, while conduct problems, hyperactivity/inattention and peer problems showed smaller but statistically significant reductions. Prosocial behavior increased slightly but significantly. Detailed results for all SDQ scales and informants are presented in Table 2.

**Table 2:** Pre- to post-treatment changes in SDQ scores

Informant	Scale	*n*	*p* value	r_rb (95% CI)
Patient	Total difficulties Emotional symptoms Conduct problems Hyperactivity/inattention Peer problems Prosocial behavior	881 881 881 881 881 881	< 0.001 < 0.001 < 0.001 < 0.001 < 0.001 < 0.001	−0.635 (− 0.681, − 0.590) −0.651 (− 0.697, − 0.606) −0.457 (− 0.524, − 0.390) −0.370 (− 0.438, − 0.302) −0.369 (− 0.438, − 0.301) 0.373 (0.307, 0.438)
Parent	Total difficulties Emotional symptoms Conduct problems Hyperactivity/inattention Peer problems Prosocial behavior	761 761 761 761 761 761	< 0.001 < 0.001 < 0.001 < 0.001 < 0.001 0.033	−0.626 (− 0.675, − 0.576) −0.649 (− 0.699, − 0.600) −0.448 (− 0.519, − 0.377) −0.392 (− 0.465, − 0.319) −0.270 (− 0.356, − 0.185) 0.103 (0.022, 0.184)

Wilcoxon signed-rank tests; rank-biserial correlations. Negative r values indicate reductions in difficulties; positive values indicate increases in prosocial behavior

*n*  number of paired cases with valid pre and post/follow-up SDQ scale scores

Exploratory analyses examined whether pre-post changes in SDQ total difficulties differed between diagnostic groups. For patient-reported outcomes, a statistically significant group difference was observed, but the associated effect size was trivial (Kruskal-Wallis H = 12.40, *p* = .030, ε² = 0.007). No significant group difference was found for parent-reported outcomes (H = 11.63, *p* = .071, ε² = 0.008), indicating no clinically meaningful moderation by diagnosis.

**Fig. 2:**
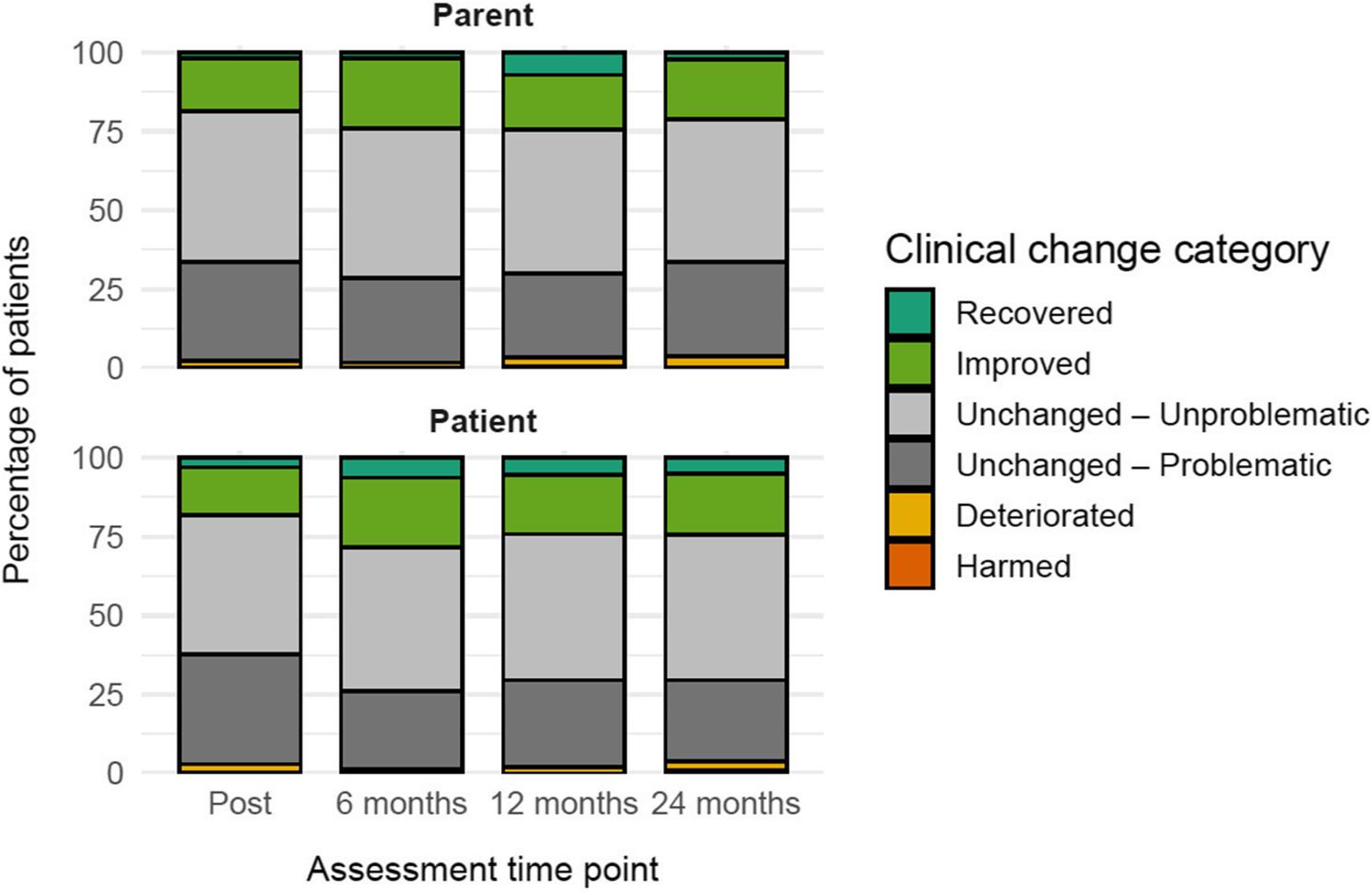
Clinically significant change in SDQ total difficulties at post-treatment and follow-up. Note. Percentages refer to patients classified as recovered, improved, unchanged, deteriorated or harmed based on reliable change criteria applied to SDQ total difficulties scores, evaluated separately for each assessment time point relative to baseline. The unchanged category is further differentiated into unchanged-unproblematic (baseline SDQ total difficulties below the clinical cutoff (17)) and unchanged-problematic (baseline SDQ total difficulties at or above the clinical cutoff). Results are shown separately for parent- and patient-reported outcomes at post-treatment and at 6-, 12- and 24-month follow-ups

Figure 2 displays the distribution of clinically significant change categories for parent- and patient-reported SDQ total difficulties. Across informants and assessment points, a considerable proportion of patients was classified as unchanged according to reliable change criteria. As illustrated, this group was predominantly composed of patients who already scored below the clinical SDQ cutoff (17) at baseline and were therefore classified as unchanged-unproblematic. In contrast, unchanged-problematic cases constituted a smaller subgroup, indicating persistent clinically elevated symptom levels.

### Pre-treatment to follow-up changes (H2)

Reductions in SDQ scores observed at post-treatment were also present at follow-up assessments conducted 6, 12 and 24 months after treatment completion. Effect sizes for total difficulties remained moderate to large across all follow-up assessments, including the 24-month follow-up, with smaller but consistent effects observed for the problem-related subscales. In parent-reported data, total difficulties scores at all follow-up time points were significantly lower than pre-treatment scores. Emotional symptoms showed a comparable pattern. Conduct problems and hyperactivity/inattention remained reduced relative to baseline, although with smaller effect sizes than those observed for emotional symptoms. Peer problems also remained lower than at pre-treatment. Prosocial behavior scores at follow-up were higher than before treatment. In patient-reported data, total difficulties and emotional symptoms were significantly reduced at all follow-up assessments compared to pre-treatment. Conduct problems also remained clearly lower than baseline up to 24 months after treatment. Reductions in hyperactivity/inattention and peer problems were maintained over time, though effects were smaller. Prosocial behavior showed sustained increases across all follow-up assessments.

For both informants, effect sizes for total difficulties remained statistically significant at all follow-up time points (Table 3). Subscale results showed the same pattern (Supplementary Tables S2 and S3).

**Table 3:** Pre-treatment to follow-up changes in SDQ total difficulties

Informant	Follow-up	*n*	*p* value	r_rb (95% CI)
Patient	FU6 FU12 FU24	363 319 254	< 0.001 < 0.001 < 0.001	−0.772 (− 0.820, − 0.725) −0.597 (− 0.678, − 0.517) −0.688 (− 0.762, − 0.615)
Parent	FU6 FU12 FU24	334 266 190	< 0.001 < 0.001 < 0.001	−0.771 (− 0.821, − 0.721) −0.619 (− 0.704, − 0.533) −0.590 (− 0.697, − 0.484)

Comparisons are pre-treatment vs. respective follow-up

*FU*  follow-up (6, 12, 24)

### Associations between SDQ outcomes and follow-up ratings (H3)

SDQ total difficulties scores at follow-up were associated with subjective ratings of treatment outcome after controlling for baseline SDQ scores. Associations were generally small to moderate for ratings of expectations fulfilled and perceived helpfulness and moderate to large for ratings referring to problem recurrence and perceived change. Descriptive trajectories of follow-up ratings are shown in Supplementary Figure S1. In parent ratings, higher SDQ scores were associated with less favourable evaluations of treatment outcome across all follow-up assessments. Associations were strongest for ratings referring to problem recurrence and perceived change compared to before treatment. In patient ratings, SDQ scores were also significantly related to follow-up ratings. Associations were smaller for ratings of expectations fulfilled and perceived helpfulness and larger for ratings addressing symptom change and problem recurrence. Associations tended to be strongest at the 24-month follow-up. Overall follow-up ratings indicated generally favourable evaluations of treatment outcome across informants and follow-up time points, with low mean values reflecting low levels of perceived problem recurrence and sustained improvement (Supplementary Table S4). Results of the partial Spearman correlation analyses are shown in Table 4.

**Table 4:** Associations between SDQ outcomes and self-rated treatment success

Informant	Follow-up	Rating	*n*	*ρ*	*p* value
Patient	FU6 FU6 FU6 FU6 FU6 FU12 FU12 FU12 FU24 FU24 FU24	Expectations fulfilled Helpfulness Problem recurrence Change vs. before treatment Change vs. end of treatment Problem recurrence Change vs. before treatment Change vs. end of treatment Problem recurrence Change vs. before treatment Change vs. end of treatment	327 327 327 327 327 304 304 304 229 229 229	0.273 0.276 0.464 0.410 0.350 0.427 0.401 0.337 0.504 0.459 0.439	< 0.001 < 0.001 < 0.001 < 0.001 < 0.001 < 0.001 < 0.001 < 0.001 < 0.001 < 0.001 < 0.001
Parent	FU6 FU6 FU6 FU6 FU6 FU12 FU12 FU12 FU24 FU24 FU24	Expectations fulfilled Helpfulness Problem recurrence Change vs. before treatment Change vs. end of treatment Problem recurrence Change vs. before treatment Change vs. end of treatment Problem recurrence Change vs. before treatment Change vs. end of treatment	268 268 268 268 268 258 258 258 154 154 154	0.416 0.433 0.493 0.462 0.323 0.406 0.415 0.351 0.585 0.568 0.505	< 0.001 < 0.001 < 0.001 < 0.001 < 0.001 < 0.001 < 0.001 < 0.001 < 0.001 < 0.001 < 0.001

Partial Spearman correlations controlling for baseline SDQ. Items expectations fulfilled and helpfulness were reverse-coded so that higher values indicate less favourable outcomes; items problem recurrence and both change items were already coded in this direction. Thus, positive correlations indicate that higher SDQ difficulties are associated with worse self-rated success

### Sensitivity analyses

Analyses using Kendall’s tau-b yielded similar patterns of association (see Supplementary Table S5). Ordinal regression analyses further showed that change in SDQ scores predicted follow-up ratings while controlling for baseline SDQ scores in both patient and parent reports (see Supplementary Table S6).

To examine potential selective dropout at long-term follow-up, baseline SDQ total difficulties scores at pre-treatment were compared between participants who did versus did not provide data at the 6-, 12- and 24-month follow-ups, separately for parent- and patient-reported outcomes. Across most comparisons, baseline SDQ scores did not differ significantly between participants with and without follow-up data and effect sizes were small, indicating little evidence for systematic selective dropout with respect to initial symptom severity. One exception was observed for parent-reported outcomes at the 12-month follow-up, where participants who provided FU12 data had slightly lower baseline SDQ total difficulties scores than those who did not (*p* = .002). However, the corresponding effect size was small, suggesting limited practical relevance.

Sensitivity analyses restricted to participants with SDQ total difficulties scores in the clinical range at baseline (≥ 17) yielded results consistent with the main analyses. Effect sizes remained large across all outcomes and time points for both patient- and parent-reported measures and were comparable or slightly larger than those observed in the full sample (see Supplementary Tables S7–S8).

## Discussion

The present study examined symptom change and long-term outcomes following CBT delivered under routine outpatient care conditions to children and adolescents. Using routinely collected outcome monitoring data, significant reductions in emotional and behavioral difficulties were observed from pre- to post-treatment in both patient- and parent-reported SDQ scores. Symptom reductions were not limited to the end of treatment. Across informants, SDQ total difficulties scores at follow-ups remained lower than pre-treatment levels. The comparatively large pre-follow-up effect sizes likely reflect cumulative symptom change over longer time intervals rather than additional treatment effects beyond the end of therapy. This pattern was particularly pronounced for emotional symptoms, while reductions in conduct problems, hyperactivity/inattention and peer problems were smaller but remained detectable at follow-up. Analyses of selective dropout provided little evidence that these effects were driven by systematic differences in baseline symptom severity between participants with and without follow-up data. Prosocial behavior showed increases following treatment and remained elevated at follow-up, especially in patient reports. Although the SDQ does not allow conclusions about specific mechanisms, the observed increases in prosocial behavior may reflect improvements in everyday social functioning accompanying symptom reduction in routine CBT. Importantly, these findings extend previous routine care evidence by indicating that treatment-related changes are not restricted to internalizing symptoms, but also encompass domains relevant to externalizing and social functioning. The relatively high proportion of unchanged cases observed in the clinically significant change analyses (Fig. 2) reflects the conservative nature of reliable change criteria when applied to broad screening instruments with a limited reliability such as the SDQ. A previous study on routine care in adolescents using the CBCL found that more than 50% of the patients showed no reliable change with about half of these in the clinical and the other half of these in the normal range post-treatment [10]. Importantly, a substantial share of unchanged cases consisted of patients who already scored below the clinical cutoff at baseline and therefore did not meet criteria for clinically significant improvement despite showing stable or low symptom levels over time. While further research is needed to address treatment non-response, this pattern of results suggests that the high number of participants classified as non-responders in this analysis may also reflect limitations in the sensitivity of the measure. Importantly, sensitivity analyses restricted to participants with clinically elevated baseline symptoms yielded comparable or slightly larger effect sizes, indicating that the observed improvements were not driven by participants with subclinical baseline scores.

This interpretation reflects the combined criteria of reliable change and movement relative to clinical thresholds, which jointly determine classification of clinically significant improvement.

The magnitude of the observed pre-post and pre-follow-up effects is comparable to those reported in other routine care studies using standardized symptom measures [5, 10]. While randomized controlled trials conducted under more controlled conditions may report effect sizes that differ from those observed in routine care, differences in study design, patient selection and treatment delivery limit direct comparability between efficacy and routine care studies [5]. Taken together, the present results are consistent with previous findings on sustained symptom reductions following CBT in youth under routine care conditions.

Emotional symptoms showed larger and more stable reductions than conduct problems, hyperactivity/inattention and peer problems. This pattern corresponds to findings from other routine care studies, which typically report stronger effects for internalizing symptoms than for externalizing domains [5, 10]. A possible explanation is that emotional symptoms are more directly addressed by CBT components commonly used in routine outpatient care, such as cognitive restructuring and emotion regulation strategies. In contrast, externalizing and peer-related difficulties are often embedded in broader interpersonal and contextual processes, including family interactions and school-related factors. These domains may therefore be less responsive to individual outpatient CBT alone. Given the cross-diagnostic and observational design of the present study, this interpretation should be viewed as tentative and not as evidence of differential treatment efficacy. In addition, the observed increases in prosocial behavior suggest that symptom reduction may be accompanied by changes in everyday social functioning. Such changes may represent a clinically relevant aspect of treatment outcome that is not fully captured by symptom severity alone. At the same time, these results highlight the need for further studies and treatment approaches that more explicitly target externalizing symptoms and contextual factors beyond individual outpatient CBT.

Exploratory analyses provided little evidence that treatment-related symptom change differed meaningfully between diagnostic groups. This pattern suggests that observed symptom improvements were broadly comparable across diagnostic categories, supporting the interpretation of cross-diagnostic effectiveness of CBT in routine outpatient care.

Standardized symptom outcomes were systematically related to participants’ subjective evaluations of treatment success. Ratings addressing problem recurrence and perceived change showed the strongest associations with SDQ scores, whereas ratings of expectations fulfilled and helpfulness were less closely related to symptom severity. This pattern suggests that different follow-up ratings reflect partially overlapping, but not identical aspects of perceived treatment success.

Importantly, subjective follow-up ratings were analysed both descriptively and in relation to standardized symptom outcomes; the latter does not imply that symptom measures represent a gold standard, but rather allows examination of overlap and divergence between different perspectives on treatment success. Considering both symptom-based measures and global outcome ratings may provide a more comprehensive picture of long-term treatment success in routine care. These findings raise the question of whether sustained improvements after treatment completion may, in some cases, reflect that symptom stabilization can occur without ongoing intervention. This interpretation remains speculative and cannot be addressed with the present data but highlights an important question for future research regarding the timing and criteria for treatment termination in routine care.

From a methodological perspective, the present study can be located on a continuum between efficacy and effectiveness research. Although CBT was delivered under routine outpatient care conditions, treatment occurred in a university-based outpatient training clinic with regular supervision and structured outcome monitoring. According to pragmatic-explanatory frameworks such as PRECIS [22] and conceptual distinctions between efficacy and effectiveness research [23], these design characteristics (e.g., practitioner expertise, follow-up intensity and outcome assessment) place the study closer to the effectiveness end of the spectrum, while maintaining elements that enhance internal validity. This intermediate positioning has important implications for interpretation. It allows conclusions regarding the real-world effectiveness of CBT under typical clinical conditions, both at the group as well as the individual level, while supporting internal validity through structured supervision and monitoring. At the same time, outcomes observed in less structured outpatient settings may differ and effect sizes should therefore not be interpreted as directly transferable to less standardized routine care contexts.

Several limitations should be considered. As this was an observational study without a control group, causal conclusions are limited and symptom changes cannot be attributed exclusively to treatment effects. In addition, although the present study was conducted under routine care conditions, treatment was delivered in a university-based outpatient training clinic, where CBT is provided under regular supervision. While supervision is common in training settings, this may limit the generalizability of the findings to routine outpatient care without structured supervision. More generally, routine outpatient care and randomized controlled trials differ along multiple dimensions, including patient selection, treatment delivery and level of supervision, rather than representing discrete categories. Improvements may in part reflect spontaneous remission, developmental changes or regression to the mean.

Participation declined across follow-up assessments, which may limit the generalizability of long-term findings. Although analyses revealed little evidence for systematic baseline differences between participants with and without follow-up data, selective participation cannot be entirely ruled out. In addition, follow-up availability depended partly on calendar time since treatment completion, such that some participants had not yet reached later follow-up time points within the observation period. These factors should be considered when interpreting long-term outcomes.

Part of the observation period coincided with the COVID-19 pandemic. Follow-up outcomes may therefore have been affected by external factors, including changes in daily routines and social restrictions. Results from this period should be interpreted cautiously when generalizing beyond pandemic conditions.

Consistent with this diagnostically heterogeneous sample, outcomes were measured using the SDQ as a broad screening instrument. As a result, disorder-specific conclusions are not possible and diagnostic subgroups were not examined separately. The term cross-diagnostic in the present study refers to the inclusion of a heterogeneous routine care sample rather than to formal comparisons between diagnostic subgroups.

Due to the use of routinely collected data, detailed information on treatment characteristics (e.g., specific interventions, session content or number of sessions) and diagnostic procedures was not available. This limits the ability to draw conclusions about specific treatment components or mechanisms of change. Furthermore, information on pharmacological treatment was not available. As a result, it was not possible to examine potential differences between CBT alone and CBT combined with medication. This represents a limitation, as medication use may have influenced symptom trajectories and long-term outcomes. Importantly, the absence of medication data reflects a common limitation of routine outcome monitoring datasets and does not indicate that pharmacological treatment was absent in the sample. This limitation should also be interpreted in the context of the German healthcare system, where pharmacological treatment in children and adolescents is typically embedded in multimodal approaches and overall prescription rates remain comparatively low. Population-based studies report prevalence rates well below 5%, depending on the definition and data source, with estimates ranging from below 1% in population surveys to approximately 2–3% in health insurance data [24, 25].

Future studies combining routine outcome monitoring with systematic treatment documentation are needed to disentangle these effects.

Within these limitations, the findings suggest that CBT delivered under routine outpatient care conditions is associated with symptom reductions that extend beyond the end of treatment. The associations between standardized symptom measures and subjective follow-up ratings suggest that symptom change is reflected in patients’ and caregivers’ evaluations of treatment outcome.

At the same time, the observed overlap is likely driven primarily by ratings that directly refer to symptom change and problem recurrence. For more general items such as perceived helpfulness or fulfilled expectations, it remains unclear which aspects of change respondents had in mind. Standardized symptom measures may therefore capture only part of what patients and caregivers consider relevant treatment effects. Qualitative analyses of patients’ and caregivers’ own descriptions of helpful changes may help to clarify these aspects and to identify potential gaps between symptom-focused outcome assessment and subjective treatment expectations.

From a clinical perspective, these findings suggest that brief, standardized transdiagnostic symptom measures are well suited for use in follow-up assessments to monitor symptom trajectories and potential relapse in routine care. At the same time, the inclusion of short global outcome ratings may provide additional clinically relevant information on patients’ and caregivers’ perceived treatment success beyond symptom change alone.

## Conclusions

Routine outpatient CBT for children and adolescents was associated with meaningful improvements in SDQ symptom burden from pre- to post-treatment that persisted up to 24 months, although clinically significant change criteria indicated more conservative individual-level outcomes. Subjective follow-up ratings were consistently associated with SDQ outcomes even after controlling for baseline severity, indicating that symptom changes were reflected in patients’ and caregivers’ evaluations of treatment success.

However, follow-up ratings suggest that the degree of correspondence between perceived treatment success and standardized symptom change varies across outcome dimensions. While ratings directly referring to symptom change and problem recurrence showed substantial overlap with SDQ outcomes, more general evaluations such as perceived helpfulness or fulfilled expectations were less closely related to symptom severity. Analyses of patients’ and caregivers’ own descriptions of helpful changes may therefore provide complementary information beyond standardized symptom measures.

## Supplementary Information

Supplementary Material 1.

## Data Availability

The data that support the findings of this study are not publicly available due to ethical and legal restrictions related to patient confidentiality, as they were collected as part of routine clinical care. Anonymized R analysis scripts will be made publicly available on PsychArchives after study completion.

## Abbreviations

CBT: Cognitive behavioral therapy
SDQ: Strengths and Difficulties Questionnaire
FU: Follow-up (FU6/FU12/FU24)
r_rb: Rank-biserial correlation

## References

[1] Arnberg A, Öst LG. CBT for Children with Depressive Symptoms: A Meta-Analysis. Cogn Behav Ther. 2014. 10.1080/16506073.2014.947316. 25248459 10.1080/16506073.2014.947316

[2] Ishikawa SI, Okajima I, Matsuoka H, Sakano Y. Cognitive Behavioural Therapy for Anxiety Disorders in Children and Adolescents: A Meta-Analysis. Child Adolesc Mental Health. 2007;12:164–72. 10.1111/j.1475-3588.2006.00433.x. 10.1111/j.1475-3588.2006.00433.x 32811007

[3] Oud M, de Winter L, Vermeulen-Smit E, Bodden D, Nauta M, Stone L, et al. Effectiveness of CBT for children and adolescents with depression: A systematic review and meta-regression analysis. Eur Psychiatry. 2019;57:33–45. 10.1016/j.eurpsy.2018.12.008. 30658278 10.1016/j.eurpsy.2018.12.008

[4] Jaite C, Seidel A, Hoffmann F, Mattejat F, Bachmann CJ. Guideline-Based Psychotherapy of Children and Adolescents in Germany: Frequency, Treatment Modalities, and Duration of Treatment. Deutsches Ärzteblatt international. 2022;119:132. 10.3238/arztebl.m2022.0106. 35506294 10.3238/arztebl.m2022.0106 PMC9160420

[5] Wergeland GJH, Riise EN, Öst L-G. Cognitive behavior therapy for internalizing disorders in children and adolescents in routine clinical care: A systematic review and meta-analysis. Clin Psychol Rev. 2021;83:101918. 10.1016/j.cpr.2020.101918. 33186776 10.1016/j.cpr.2020.101918

[6] Wuthrich VM, Zagic D, Dickson SJ, McLellan LF, Chen JT-H, Jones MP, Rapee RM. Effectiveness of Psychotherapy for Internalising Symptoms in Children and Adolescents When Delivered in Routine Settings: A Systematic Review and Meta-analysis. Clin Child Fam Psychol Rev. 2023;26:824–48. 10.1007/s10567-023-00433-8. 37059918 10.1007/s10567-023-00433-8 PMC10465434

[7] Walter D, Behrendt U, Matthias E, Hellmich M, Dachs L, Goletz H, Doepfner M, et al. Effectiveness and long-term stability of outpatient cognitive behavioural therapy (CBT) for children and adolescents with anxiety and depressive disorders under routine care conditions. Behav Cogn Psychother. 2023;51(4):320–34. 10.1017/S1352465823000073. 36908248 10.1017/S1352465823000073

[8] Krause K, Zhang XC, Schneider S. Long-Term Effectiveness of Cognitive Behavioral Therapy in Routine Outpatient Care for Youth with Anxiety Disorders. Psychother Psychosom. 2024;93:181–90. 10.1159/000537932. 38615662 10.1159/000537932 PMC11151973

[9] von Brachel R, Hirschfeld G, Berner A, Willutzki U, Teismann T, Cwik JC, et al. Long-Term Effectiveness of Cognitive Behavioral Therapy in Routine Outpatient Care: A 5- to 20-Year Follow-Up Study. Psychother Psychosom. 2019;88:225–35. 10.1159/000500188. 31121580 10.1159/000500188

[10] Walter D, Dachs L, Faber M, Goletz H, Goertz-Dorten A, Hautmann C, et al. Effectiveness of outpatient cognitive-behavioral therapy for adolescents under routine care conditions on behavioral and emotional problems rated by parents and patients: an observational study. Eur Child Adolesc Psychiatry. 2018;27:65–77. 10.1007/s00787-017-1021-z. 28685400 10.1007/s00787-017-1021-z

[11] Walter D, Dachs L, Faber M, Goletz H, Görtz-Dorten A, Kinnen C, Döpfner M, et al. Alltagswirksamkeit ambulanter Verhaltenstherapie von Kindern und Jugendlichen im Urteil der Eltern in einer universitären Ausbildungsambulanz. Z für Klinische Psychologie und Psychother. 2015. 10.1026/1616-3443/a000321.

[12] Michalak J, Kosfelder J, Meyer F, Schulte D. Messung des Therapieerfolgs. Z für Klinische Psychologie und Psychother. 2003. 10.1026/0084-5345.32.2.94.

[13] Kwan B, Dimidjian S, Rizvi SL. Treatment preference, engagement, and clinical improvement in pharmacotherapy versus psychotherapy for depression. Behav Res Ther. 2010;48:799–804. 10.1016/j.brat.2010.04.003. 20462569 10.1016/j.brat.2010.04.003 PMC2918721

[14] Jacobson NS, Truax P. Clinical significance: a statistical approach to defining meaningful change in psychotherapy research. J Consult Clin Psychol. 1991;59(1):12–9. 10.1037/0022-006X.59.1.12. 2002127 10.1037//0022-006x.59.1.12

[15] Schneider S, Pflug V, In. In: Albon T, Margraf J, editors. Kinder-DIPS: Diagnostisches Interview bei psychischen Störungen im Kindes- und Jugendalter. Berlin: Springer; 2017. 10.13154/rub.101.90. -.

[16] In-Albon T, Christiansen H, Imort S, Krause K, Schlarb A, Schneider S, Schwarz D, Weber L, Velten J, Forschungsnetzwerk KODAP. Pilotdaten zur Inanspruchnahmepopulation universitärer Psychotherapie-Ambulanzen für Kinder und Jugendliche. Zeitschrift für Klinische Psychologie und Psychotherapie. 2019. 10.1026/1616-3443/a000528

[17] Margraf J, Hoyer J, Fydrich T, In-Albon T, Lincoln T, Lutz W, Schlarb A, Schöttke H, Willutzki U, Velten J. The cooperative revolution reaches clinical psychology and psychotherapy: An example from Germany. Clin Psychol Europe. 2021;3(1):e4459. 10.32872/cpe.4459. 10.32872/cpe.4459 PMC9667120 36397785

[18] Lohbeck A, Schultheiß J, Petermann F, Petermann U. Die deutsche Selbstbeurteilungsversion des Strengths and Difficulties Questionnaire (SDQ-Deu-S). Diagnostica 2015. 10.1026/0012-1924/a000153

[19] Claus BB, Wager J, Bonnet U. Clinical significance analyses of intervention studies in R: The clinicalsignificance package. J Stat Softw. 2024;111:1–39. 10.18637/jss.v111.i01.

[20] Ben-Shachar MS, Lüdecke D, Makowski D, Effectsize. Estimation of effect size indices and standardized parameters. J Open Source Softw. 2020;5:2815. 10.21105/joss.02815.

[21] Christensen RHB. ordinal: Regression models for ordinal data (R package version 2023.12–4.1). Vienna: R Foundation for Statistical Computing; 2023. https://cran.r-project.org/web/packages/ordinal/ordinal.pdf.

[22] Thorpe KE, Zwarenstein M, Oxman AD, Treweek S, Furberg CD, Altman DG, Chalkidou K, et al. A pragmatic–explanatory continuum indicator summary (PRECIS): a tool to help trial designers. J Clin Epidemiol. 2009;62(5):464–75. 10.1016/j.jclinepi.2008.12.011. 19348971 10.1016/j.jclinepi.2008.12.011

[23] Singal AG, Higgins PD, Waljee AK. A primer on effectiveness and efficacy trials. Clin Transl Gastroenterol. 2014;5(1):e45. 10.1038/ctg.2013.13. 24384867 10.1038/ctg.2013.13 PMC3912314

[24] Abbas S, Ihle P, Adler JB, Engel S, Günster C, Linder R, et al. Psychopharmacological prescriptions in children and adolescents in Germany: a nationwide analysis of over 4 million statutorily insured individuals from 2004 to 2012. Dtsch Arztebl Int. 2016;113(22–23):396–403. 10.3238/arztebl.2016.0396. 27374377 10.3238/arztebl.2016.0396 PMC4933807

[25] Koelch M, Prestel A, Singer H, Keller F, Fegert JM, Schlack R, et al. Psychotropic medication in children and adolescents in Germany: prevalence, indications, and psychopathological patterns. J Child Adolesc Psychopharmacol. 2009;19(6):765–70. 10.1089/cap.2009.0018. 20035595 10.1089/cap.2009.0018

