# Supplementary material for: Long-term effectiveness of cognitive behavioral therapy (CBT) for children and adolescents in routine care
Source: BMC Psychol. 2026 May 6;14:663. doi: 10.1186/s40359-026-04667-3 (PMC13147788; doi:10.1186/s40359-026-04667-3)
Supplement: Supplementary file 1 — Supplementary Material 1. [file 40359_2026_4667_MOESM1_ESM.docx]

**Supplementary Material**

**Supplementary Figure S1**

*Ordinal follow-up ratings (descriptive)*


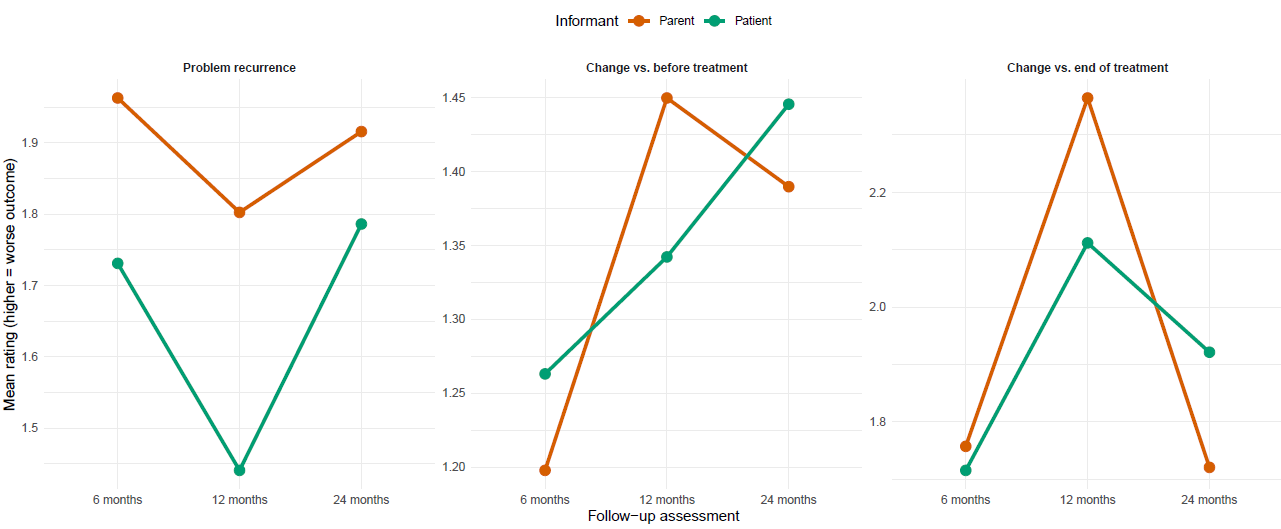


*Note.* Descriptive means of ordinal follow-up ratings are shown separately for parent- and patient-reported outcomes at 6-, 12-, and 24-month follow-ups. Higher values indicate less favourable outcomes. Values are based on analysis-specific subsamples available at each follow-up.

**Supplementary Table S1**

*Descriptive statistics of SDQ scores based on analysis-specific paired samples*

| **Infor-mant** | **Compari-son** | **SDQ scale** | | **n** | **Mean Pre** | **SD Pre** | **Mean Post/FU** | **SD Post/FU** |
| --- | --- | --- | --- | --- | --- | --- | --- | --- |
| Patient | Pre-Post | Total difficulties | 881 | | 16.25 | 5.68 | 13.14 | 6.02 |
| Patient | Pre-Post | Emotional symptoms | 881 | | 5.79 | 2.76 | 4.35 | 2.76 |
| Patient | Pre-Post | Conduct problems | 881 | | 2.36 | 1.69 | 1.82 | 1.54 |
| Patient | Pre-Post | Hyperactivity/inatt. | 881 | | 4.54 | 2.29 | 3.93 | 2.29 |
| Patient | Pre-Post | Peer problems | 881 | | 3.56 | 2.07 | 3.04 | 1.99 |
| Patient | Pre-Post | Prosocial behavior | 881 | | 7.88 | 1.91 | 8.33 | 1.81 |
| Patient | Pre-FU6 | Total difficulties | 363 | | 15.69 | 5.48 | 11.18 | 5.73 |
| Patient | Pre-FU6 | Emotional symptoms | 363 | | 5.56 | 2.81 | 3.61 | 2.80 |
| Patient | Pre-FU6 | Conduct problems | 363 | | 2.36 | 1.57 | 1.47 | 1.46 |
| Patient | Pre-FU6 | Hyperactivity/inatt. | 363 | | 4.32 | 2.26 | 3.24 | 2.23 |
| Patient | Pre-FU6 | Peer problems | 363 | | 3.44 | 1.99 | 2.85 | 1.89 |
| Patient | Pre-FU6 | Prosocial behavior | 363 | | 7.90 | 1.84 | 8.52 | 1.66 |
| Patient | Pre-FU12 | Total difficulties | 319 | | 15.66 | 5.65 | 12.24 | 6.29 |
| Patient | Pre-FU12 | Emotional symptoms | 319 | | 5.60 | 2.80 | 4.10 | 2.91 |
| Patient | Pre-FU12 | Conduct problems | 319 | | 2.31 | 1.60 | 1.51 | 1.42 |
| Patient | Pre-FU12 | Hyperactivity/inatt. | 319 | | 4.43 | 2.28 | 3.72 | 2.40 |
| Patient | Pre-FU12 | Peer problems | 319 | | 3.32 | 1.94 | 2.91 | 1.98 |
| Patient | Pre-FU12 | Prosocial behavior | 319 | | 7.81 | 1.90 | 8.54 | 1.65 |
| Patient | Pre-FU24 | Total difficulties | 254 | | 15.83 | 5.45 | 11.75 | 6.14 |
| Patient | Pre-FU24 | Emotional symptoms | 254 | | 5.62 | 2.66 | 3.93 | 2.99 |
| Patient | Pre-FU24 | Conduct problems | 254 | | 2.28 | 1.58 | 1.38 | 1.42 |
| Patient | Pre-FU24 | Hyperactivity/inatt. | 254 | | 4.46 | 2.24 | 3.57 | 2.54 |
| Patient | Pre-FU24 | Peer problems | 254 | | 3.47 | 2.02 | 2.87 | 1.88 |
| Patient | Pre-FU24 | Prosocial behavior | 254 | | 7.82 | 1.95 | 8.58 | 1.64 |
| Parent | Pre-Post | Total difficulties | 761 | | 15.77 | 6.02 | 12.68 | 6.37 |
| Parent | Pre-Post | Emotional symptoms | 761 | | 5.34 | 2.57 | 3.82 | 2.58 |
| Parent | Pre-Post | Conduct problems | 761 | | 3.01 | 2.08 | 2.43 | 1.92 |
| Parent | Pre-Post | Hyperactivity/inatt. | 761 | | 4.54 | 2.82 | 3.91 | 2.59 |
| Parent | Pre-Post | Peer problems | 761 | | 2.88 | 2.15 | 2.52 | 2.14 |
| Parent | Pre-Post | Prosocial behavior | 761 | | 7.39 | 1.96 | 7.55 | 1.91 |
| Parent | Pre-FU6 | Total difficulties | 334 | | 15.51 | 6.25 | 11.33 | 6.42 |
| Parent | Pre-FU6 | Emotional symptoms | 334 | | 5.12 | 2.57 | 3.12 | 2.45 |
| Parent | Pre-FU6 | Conduct problems | 334 | | 3.02 | 2.08 | 2.23 | 1.95 |
| Parent | Pre-FU6 | Hyperactivity/inatt. | 334 | | 4.54 | 2.90 | 3.51 | 2.59 |
| Parent | Pre-FU6 | Peer problems | 334 | | 2.82 | 2.16 | 2.47 | 2.16 |
| Parent | Pre-FU6 | Prosocial behavior | 334 | | 7.34 | 1.93 | 7.66 | 1.95 |
| Parent | Pre-FU12 | Total difficulties | 266 | | 14.74 | 5.86 | 11.29 | 7.13 |
| Parent | Pre-FU12 | Emotional symptoms | 266 | | 4.83 | 2.49 | 3.25 | 2.74 |
| Parent | Pre-FU12 | Conduct problems | 266 | | 2.92 | 1.94 | 2.29 | 1.91 |
| Parent | Pre-FU12 | Hyperactivity/inatt. | 266 | | 4.29 | 2.88 | 3.42 | 2.75 |
| Parent | Pre-FU12 | Peer problems | 266 | | 2.70 | 2.22 | 2.32 | 2.37 |
| Parent | Pre-FU12 | Prosocial behavior | 266 | | 7.43 | 1.88 | 7.73 | 1.84 |
| Parent | Pre-FU24 | Total difficulties | 190 | | 15.27 | 6.31 | 11.57 | 7.34 |
| Parent | Pre-FU24 | Emotional symptoms | 190 | | 4.83 | 2.49 | 3.27 | 2.78 |
| Parent | Pre-FU24 | Conduct problems | 190 | | 3.11 | 2.06 | 2.35 | 2.06 |
| Parent | Pre-FU24 | Hyperactivity/inatt. | 190 | | 4.62 | 2.78 | 3.67 | 2.83 |
| Parent | Pre-FU24 | Peer problems | 190 | | 2.71 | 2.27 | 2.29 | 2.06 |
| Parent | Pre-FU24 | Prosocial behavior | 190 | | 7.27 | 2.07 | 7.71 | 1.92 |

*Note.* Higher scores on SDQ problem scales indicate greater difficulties; higher scores on the prosocial scale indicate more prosocial behavior.

**Supplementary Table S2**

*Pre-treatment to follow-up changes in patient-reported SDQ subscales*

| **Follow-up** | **Scale** | **n** | **p value** | **r_rb (95% CI)** |
| --- | --- | --- | --- | --- |
| FU6 | Total difficulties  Emotional symptoms  Conduct problems  Hyperactivity/inattention  Peer problems  Prosocial behavior | 363  363  363  363  363  363 | < .001  < .001  < .001  < .001  < .001  < .001 | −0.772 (−0.820, −0.725)  −0.761 (−0.814, −0.708)  −0.661 (−0.739, −0.583)  −0.586 (−0.668, −0.504)  −0.389 (−0.493, −0.284)  0.442 (0.347, 0.537) |
| FU12 | Total difficulties  Emotional symptoms  Conduct problems  Hyperactivity/inattention  Peer problems  Prosocial behavior | 319  319  319  319  319  319 | < .001  < .001  < .001  < .001  < .001  < .001 | −0.597 (−0.678, −0.517)  −0.608 (−0.692, −0.524)  −0.602 (−0.694, −0.509)  −0.369 (−0.483, −0.255)  −0.254 (−0.378, −0.129)  0.498 (0.403, 0.592) |
| FU24 | Total difficulties  Emotional symptoms  Conduct problems  Hyperactivity/inattention  Peer problems  Prosocial behavior | 254  254  254  254  254  254 | < .001  < .001  < .001  < .001  < .001  < .001 | −0.688 (−0.762, −0.615)  −0.643 (−0.732, −0.554)  −0.690 (−0.778, −0.603)  −0.417 (−0.539, −0.295)  −0.373 (−0.501, −0.245)  0.508 (0.404, 0.612) |

*Note.* Wilcoxon signed-rank tests comparing pre-treatment and follow-up scores. Effect sizes are rank-biserial correlations (r_rb) with 95% confidence intervals. Negative values indicate reductions in difficulties; positive values indicate increases in prosocial behaviour. FU = follow-up (6, 12, 24 months). n = number of paired cases with valid pre-treatment and follow-up SDQ scale scores.

**Supplementary Table S3**

*Pre-treatment to follow-up changes in parent-reported SDQ subscales*

| **Follow-up** | **Scale** | **n** | **p value** | **r_rb (95% CI)** |
| --- | --- | --- | --- | --- |
| FU6 | Total difficulties  Emotional symptoms  Conduct problems  Hyperactivity/inattention  Peer problems  Prosocial behavior | 334  334  334  334  334  334 | < .001  < .001  < .001  < .001  < .001  .002 | −0.771 (−0.821, −0.721)  −0.747 (−0.806, −0.688)  −0.576 (−0.666, −0.485)  −0.601 (−0.686, −0.517)  −0.261 (−0.390, −0.131)  0.218 (0.100, 0.335) |
| FU12 | Total difficulties  Emotional symptoms  Conduct problems  Hyperactivity/inattention  Peer problems  Prosocial behavior | 266  266  266  266  266  266 | < .001  < .001  < .001  < .001  < .001  .005 | −0.619 (−0.704, −0.533)  −0.581 (−0.682, −0.481)  −0.494 (−0.607, −0.382)  −0.534 (−0.641, −0.426)  −0.273 (−0.426, −0.120)  0.215 (0.083, 0.346) |
| FU24 | Total difficulties  Emotional symptoms  Conduct problems  Hyperactivity/inattention  Peer problems  Prosocial behavior | 190  190  190  190  190  190 | < .001  < .001  < .001  < .001  .004  .008 | −0.590 (−0.697, −0.484)  −0.549 (−0.672, −0.425)  −0.510 (−0.643, −0.377)  −0.482 (−0.615, −0.349)  −0.265 (−0.439, −0.092)  0.239 (0.086, 0.392) |

*Note.* Wilcoxon signed-rank tests comparing pre-treatment and follow-up scores. Effect sizes are rank-biserial correlations (r_rb) with 95% confidence intervals. Negative values indicate reductions in difficulties; positive values indicate increases in prosocial behaviour. FU = follow-up (6, 12, 24 months). n = number of paired cases with valid pre-treatment and follow-up SDQ scale scores.

**Supplementary Table S4**

*Descriptive statistics of self-rated treatment-success ratings at follow-up*

| **Informant** | **FU** | **Rating** | **n** | **Mean** | **SD** |
| --- | --- | --- | --- | --- | --- |
| Parent | FU6 | Expectations fulfilled | 268 | 1.53 | 1.20 |
| Parent | FU6 | Helpfulness | 268 | 1.60 | 1.19 |
| Parent | FU6 | Problem recurrence | 268 | 1.96 | 1.37 |
| Parent | FU6 | Change vs. before treatment | 268 | 1.20 | 1.04 |
| Parent | FU6 | Change vs. end of treatment | 268 | 1.76 | 1.24 |
| Parent | FU12 | Problem recurrence | 258 | 1.80 | 1.59 |
| Parent | FU12 | Change vs. before treatment | 258 | 1.45 | 1.37 |
| Parent | FU12 | Change vs. end of treatment | 258 | 2.36 | 1.33 |
| Parent | FU24 | Problem recurrence | 154 | 1.92 | 1.44 |
| Parent | FU24 | Change vs. before treatment | 154 | 1.39 | 1.40 |
| Parent | FU24 | Change vs. end of treatment | 154 | 1.72 | 1.51 |
| Patient | FU6 | Expectations fulfilled | 327 | 1.29 | 1.17 |
| Patient | FU6 | Helpfulness | 327 | 1.23 | 1.19 |
| Patient | FU6 | Problem recurrence | 327 | 1.73 | 1.32 |
| Patient | FU6 | Change vs. before treatment | 327 | 1.26 | 1.25 |
| Patient | FU6 | Change vs. end of treatment | 327 | 1.72 | 1.38 |
| Patient | FU12 | Problem recurrence | 304 | 1.44 | 1.46 |
| Patient | FU12 | Change vs. before treatment | 304 | 1.34 | 1.24 |
| Patient | FU12 | Change vs. end of treatment | 304 | 2.11 | 1.36 |
| Patient | FU24 | Problem recurrence | 229 | 1.79 | 1.53 |
| Patient | FU24 | Change vs. before treatment | 229 | 1.45 | 1.44 |
| Patient | FU24 | Change vs. end of treatment | 229 | 1.92 | 1.50 |

*Note.* Follow-up ratings were assessed on ordinal scales and are reported as means and standard deviations for descriptive purposes. Higher values indicate less favourable outcomes. FU = follow-up (6, 12, 24 months).

**Supplementary Table S5**

*Kendall’s tau-b correlations between SDQ outcomes and follow-up ratings*

| **Informant** | **FU** | **Rating** | **SDQ metric** | **n** | **tau-b** |
| --- | --- | --- | --- | --- | --- |
| Parent | FU6 | Expectations fulfilled | Follow-up SDQ | 339 | 0.389 |
| Parent | FU6 | Helpfulness | Follow-up SDQ | 339 | 0.396 |
| Parent | FU6 | Problem recurrence | Follow-up SDQ | 339 | 0.425 |
| Parent | FU6 | Change vs. before t. | Follow-up SDQ | 339 | 0.417 |
| Parent | FU6 | Change vs. end of t. | Follow-up SDQ | 339 | 0.282 |
| Parent | FU12 | Problem recurrence | Follow-up SDQ | 353 | 0.377 |
| Parent | FU12 | Change vs. before t. | Follow-up SDQ | 353 | 0.344 |
| Parent | FU12 | Change vs. end of t. | Follow-up SDQ | 353 | 0.243 |
| Parent | FU24 | Problem recurrence | Follow-up SDQ | 210 | 0.477 |
| Parent | FU24 | Change vs. before t. | Follow-up SDQ | 210 | 0.455 |
| Parent | FU24 | Change vs. end of t. | Follow-up SDQ | 210 | 0.363 |
| Parent | FU6 | Expectations fulfilled | SDQ change | 268 | 0.151 |
| Parent | FU6 | Helpfulness | SDQ change | 268 | 0.165 |
| Parent | FU6 | Problem recurrence | SDQ change | 268 | 0.198 |
| Parent | FU6 | Change vs. before t. | SDQ change | 268 | 0.191 |
| Parent | FU6 | Change vs. end of t. | SDQ change | 268 | 0.143 |
| Parent | FU12 | Problem recurrence | SDQ change | 258 | 0.244 |
| Parent | FU12 | Change vs. before t. | SDQ change | 258 | 0.270 |
| Parent | FU12 | Change vs. end of t. | SDQ change | 258 | 0.250 |
| Parent | FU24 | Problem recurrence | SDQ change | 154 | 0.331 |
| Parent | FU24 | Change vs. before t. | SDQ change | 154 | 0.337 |
| Parent | FU24 | Change vs. end of t. | SDQ change | 154 | 0.298 |
| Patient | FU6 | Expectations fulfilled | Follow-up SDQ | 427 | 0.225 |
| Patient | FU6 | Helpfulness | Follow-up SDQ | 427 | 0.182 |
| Patient | FU6 | Problem recurrence | Follow-up SDQ | 427 | 0.378 |
| Patient | FU6 | Change vs. before t. | Follow-up SDQ | 427 | 0.287 |
| Patient | FU6 | Change vs. end of t. | Follow-up SDQ | 427 | 0.191 |
| Patient | FU12 | Problem recurrence | Follow-up SDQ | 417 | 0.333 |
| Patient | FU12 | Change vs. before t. | Follow-up SDQ | 417 | 0.299 |
| Patient | FU12 | Change vs. end of t. | Follow-up SDQ | 417 | 0.243 |
| Patient | FU24 | Problem recurrence | Follow-up SDQ | 333 | 0.393 |
| Patient | FU24 | Change vs. before t. | Follow-up SDQ | 333 | 0.374 |
| Patient | FU24 | Change vs. end of t. | Follow-up SDQ | 333 | 0.310 |
| Patient | FU6 | Expectations fulfilled | SDQ change | 327 | 0.157 |
| Patient | FU6 | Helpfulness | SDQ change | 327 | 0.190 |
| Patient | FU6 | Problem recurrence | SDQ change | 327 | 0.196 |
| Patient | FU6 | Change vs. before t. | SDQ change | 327 | 0.250 |
| Patient | FU6 | Change vs. end of t. | SDQ change | 327 | 0.217 |
| Patient | FU12 | Problem recurrence | SDQ change | 304 | 0.263 |
| Patient | FU12 | Change vs. before t. | SDQ change | 304 | 0.247 |
| Patient | FU12 | Change vs. end of t. | SDQ change | 304 | 0.194 |
| Patient | FU24 | Problem recurrence | SDQ change | 229 | 0.234 |
| Patient | FU24 | Change vs. before t. | SDQ change | 229 | 0.240 |
| Patient | FU24 | Change vs. end of t. | SDQ change | 229 | 0.259 |

*Note.* Kendall’s tau-b correlations between SDQ total difficulties (follow-up scores or change scores) and ordinal follow-up ratings. Higher values indicate worse outcomes. Sample sizes differ depending on availability of SDQ follow-up scores vs. change scores. FU = follow-up (6, 12, 24 months).

**Supplementary Table S6**

*Ordinal regression analyses predicting follow-up ratings from SDQ change*

| **Informant** | **FU** | **Rating** | **n** | **β_change** | **SE** | **z** | **p** |
| --- | --- | --- | --- | --- | --- | --- | --- |
| Parent | FU6 | Expectations fulfilled | 268 | 0.164 | 0.024 | 6.86 | < .001 |
| Parent | FU6 | Helpfulness | 268 | 0.178 | 0.025 | 7.19 | < .001 |
| Parent | FU6 | Problem recurrence | 268 | 0.208 | 0.026 | 8.06 | < .001 |
| Parent | FU6 | Change vs. before t. | 268 | 0.190 | 0.026 | 7.27 | < .001 |
| Parent | FU6 | Change vs. end of t. | 268 | 0.133 | 0.024 | 5.54 | < .001 |
| Parent | FU12 | Problem recurrence | 258 | 0.164 | 0.022 | 7.35 | < .001 |
| Parent | FU12 | Change vs. before t. | 258 | 0.160 | 0.022 | 7.14 | < .001 |
| Parent | FU12 | Change vs. end of t. | 258 | 0.138 | 0.022 | 6.26 | < .001 |
| Parent | FU24 | Problem recurrence | 154 | 0.211 | 0.029 | 7.20 | < .001 |
| Parent | FU24 | Change vs. before t. | 154 | 0.219 | 0.031 | 7.17 | < .001 |
| Parent | FU24 | Change vs. end of t. | 154 | 0.173 | 0.028 | 6.18 | < .001 |
| Patient | FU6 | Expectations fulfilled | 327 | 0.108 | 0.022 | 4.93 | < .001 |
| Patient | FU6 | Helpfulness | 327 | 0.114 | 0.022 | 5.20 | < .001 |
| Patient | FU6 | Problem recurrence | 327 | 0.195 | 0.023 | 8.41 | < .001 |
| Patient | FU6 | Change vs. before t. | 327 | 0.183 | 0.023 | 7.88 | < .001 |
| Patient | FU6 | Change vs. end of t. | 327 | 0.144 | 0.022 | 6.66 | < .001 |
| Patient | FU12 | Problem recurrence | 304 | 0.157 | 0.021 | 7.50 | < .001 |
| Patient | FU12 | Change vs. before t. | 304 | 0.145 | 0.021 | 6.91 | < .001 |
| Patient | FU12 | Change vs. end of t. | 304 | 0.119 | 0.020 | 5.82 | < .001 |
| Patient | FU24 | Problem recurrence | 229 | 0.194 | 0.025 | 7.63 | < .001 |
| Patient | FU24 | Change vs. before t. | 229 | 0.180 | 0.025 | 7.13 | < .001 |
| Patient | FU24 | Change vs. end of t. | 229 | 0.162 | 0.024 | 6.70 | < .001 |

*Note.* Ordinal regression analyses (cumulative link models) predicting ordinal follow-up ratings from SDQ change scores, controlling for baseline SDQ total difficulties.

**Supplementary Table S7**

*Pre- to post-treatment changes in SDQ scores (clinical subsample, SDQ ≥ 17 at baseline)*

| **Informant** | **Scale** | **n** | **p value** | **r_rb (95% CI)** |
| --- | --- | --- | --- | --- |
| Patient | Total difficulties | 436 | < .001 | −0.822 (−0.854, −0.784) |
| Patient | Emotional symptoms | 436 | < .001 | −0.815 (−0.849, −0.775) |
| Patient | Conduct problems | 436 | < .001 | −0.602 (−0.671, −0.523) |
| Patient | Hyperactivity/inattention | 436 | < .001 | −0.524 (−0.599, −0.440) |
| Patient | Peer problems | 436 | < .001 | −0.582 (−0.650, −0.504) |
| Patient | Prosocial behavior | 436 | < .001 | 0.403 (0.308, 0.489) |
| Parent | Total difficulties | 354 | < .001 | −0.816 (−0.853, −0.772) |
| Parent | Emotional symptoms | 354 | < .001 | −0.749 (−0.798, −0.689) |
| Parent | Conduct problems | 354 | < .001 | −0.686 (−0.747, −0.615) |
| Parent | Hyperactivity/inattention | 354 | < .001 | −0.668 (−0.730, −0.595) |
| Parent | Peer problems | 354 | < .001 | −0.445 (−0.539, −0.339) |
| Parent | Prosocial behavior | 354 | .001 | 0.236 (0.119, 0.346) |

*Note.* Analyses were restricted to participants with SDQ total difficulties scores in the clinical range at baseline (≥17). Results are based on paired Wilcoxon signed-rank tests. Effect sizes are reported as rank-biserial correlations (r_rb) with 95% confidence intervals.

**Supplementary Table S8**

*Pre- to follow-up changes in SDQ scores (clinical subsample, SDQ ≥ 17 at baseline)*

| **Informant** | **FU** | **Scale** | **n** | **p value** | **r_rb (95% CI)** |
| --- | --- | --- | --- | --- | --- |
| Patient | FU6 | Total difficulties | 167 | < .001 | −0.946 (−0.962, −0.925) |
| Patient | FU6 | Emotional symptoms | 167 | < .001 | −0.904 (−0.932, −0.865) |
| Patient | FU6 | Conduct problems | 167 | < .001 | −0.726 (−0.805, −0.622) |
| Patient | FU6 | Hyperactivity/inattention | 167 | < .001 | −0.768 (−0.833, −0.682) |
| Patient | FU6 | Peer problems | 167 | < .001 | −0.748 (−0.817, −0.658) |
| Patient | FU6 | Prosocial behavior | 167 | < .001 | 0.496 (0.354, 0.616) |
| Patient | FU12 | Total difficulties | 149 | < .001 | −0.823 (−0.874, −0.753) |
| Patient | FU12 | Emotional symptoms | 149 | < .001 | −0.859 (−0.901, −0.800) |
| Patient | FU12 | Conduct problems | 149 | < .001 | −0.718 (−0.804, −0.604) |
| Patient | FU12 | Hyperactivity/inattention | 149 | < .001 | −0.562 (−0.678, −0.419) |
| Patient | FU12 | Peer problems | 149 | < .001 | −0.450 (−0.589, −0.287) |
| Patient | FU12 | Prosocial behavior | 149 | < .001 | 0.499 (0.348, 0.625) |
| Patient | FU24 | Total difficulties | 117 | < .001 | −0.851 (−0.899, −0.782) |
| Patient | FU24 | Emotional symptoms | 117 | < .001 | −0.773 (−0.848, −0.667) |
| Patient | FU24 | Conduct problems | 117 | < .001 | −0.856 (−0.908, −0.778) |
| Patient | FU24 | Hyperactivity/inattention | 117 | < .001 | −0.598 (−0.719, −0.443) |
| Patient | FU24 | Peer problems | 117 | < .001 | −0.619 (−0.734, −0.470) |
| Patient | FU24 | Prosocial behavior | 117 | < .001 | 0.648 (0.510, 0.753) |
| Parent | FU6 | Total difficulties | 144 | < .001 | −0.911 (−0.938, −0.873) |
| Parent | FU6 | Emotional symptoms | 144 | < .001 | −0.823 (−0.878, −0.748) |
| Parent | FU6 | Conduct problems | 144 | < .001 | −0.763 (−0.833, −0.670) |
| Parent | FU6 | Hyperactivity/inattention | 144 | < .001 | −0.829 (−0.880, −0.759) |
| Parent | FU6 | Peer problems | 144 | < .001 | −0.438 (−0.583, −0.266) |
| Parent | FU6 | Prosocial behavior | 144 | .031 | 0.228 (0.044, 0.397) |
| Parent | FU12 | Total difficulties | 104 | < .001 | −0.761 (−0.840, −0.650) |
| Parent | FU12 | Emotional symptoms | 104 | < .001 | −0.620 (−0.744, −0.455) |
| Parent | FU12 | Conduct problems | 104 | < .001 | −0.645 (−0.758, −0.494) |
| Parent | FU12 | Hyperactivity/inattention | 104 | < .001 | −0.686 (−0.789, −0.545) |
| Parent | FU12 | Peer problems | 104 | < .001 | −0.446 (−0.617, −0.235) |
| Parent | FU12 | Prosocial behavior | 104 | .006 | 0.338 (0.130, 0.518) |
| Parent | FU24 | Total difficulties | 83 | < .001 | −0.761 (−0.847, −0.636) |
| Parent | FU24 | Emotional symptoms | 83 | < .001 | −0.642 (−0.773, −0.457) |
| Parent | FU24 | Conduct problems | 83 | < .001 | −0.610 (−0.748, −0.422) |
| Parent | FU24 | Hyperactivity/inattention | 83 | < .001 | −0.659 (−0.780, −0.490) |
| Parent | FU24 | Peer problems | 83 | < .001 | −0.612 (−0.752, −0.419) |
| Parent | FU24 | Prosocial behavior | 83 | .006 | 0.369 (0.139, 0.562) |

*Note.* Analyses were restricted to participants with SDQ total difficulties scores in the clinical range at baseline (≥17). Results are based on paired Wilcoxon signed-rank tests. Effect sizes are reported as rank-biserial correlations (r_rb) with 95% confidence intervals.
